# An acute care surgery service expedites the treatment of emergency colorectal cancer: a retrospective case–control study

**DOI:** 10.1186/1749-7922-9-19

**Published:** 2014-03-21

**Authors:** Ram Venkatesh Anantha, Muriel Brackstone, Neil Parry, Ken Leslie

**Affiliations:** 1 Division of General Surgery, Department of Surgery, Schulich School of Medicine and Dentistry, Western University, London, Ontario, Canada; 2 Division of Critical Care, Department of Medicine, Schulich School of Medicine and Dentistry, Western University, London, Ontario, Canada; 3 Trauma Program, London Health Sciences Centre, London, Ontario, Canada; 4 London Health Sciences Centre, Victoria Hospital, Room E2-217, 800 Commissioners Road East, London, Ontario N6A 5 W9, Canada

**Keywords:** Colorectal/anal neoplasia, Colonoscopy, Acute care surgery, Health outcomes

## Abstract

**Introduction:**

Emergency colorectal cancer (CRC) is a complex disease that requires multidisciplinary approaches for management. However, it is unclear whether acute care surgery (ACS) services can expedite the workup and treatment of complex surgical diseases such as emergency CRC. We sought to assess the impact of an Acute Care and Emergency Surgery Service (ACCESS) on wait-times for inpatient colonoscopy and surgical resection among emergency CRC patients.

**Methods:**

This retrospective case–control study was conducted at a tertiary-care, university-affiliated, cancer centre in London, Ontario, Canada. All patients aged 18 or older who presented to the emergency department with a recent (within 48 hours) diagnosis of CRC, or were diagnosed with CRC after admission, were included in the study. Patients were either in the pre-ACCESS (July 1, 2007-June 31, 2010) or post-ACCESS (July 1, 2010-June 30, 2012) groups. A third group of emergency CRC patients treated at an adjacent cancer centre that lacked ACCESS (non-ACCESS) was evaluated separately. The primary outcome was time from admission to colonoscopy and surgery.

**Results:**

A total of 149 patients (47 pre-ACCESS, 37 post-ACCESS, and 65 non-ACCESS) were identified. Only 19% (n = 9) of pre-ACCESS patients underwent inpatient colonoscopy, compared to 38% (n = 14) in the post-ACCESS group (p = 0.023). Additionally, 100% of patients in the post-ACCESS era underwent inpatient colonoscopy and surgery during the same admission, compared to only 44% of pre-ACCESS patients (p = 0.006). Median wait-times for inpatient colonoscopy (2.0 and 1.8 days for pre- and post-ACCESS groups respectively, p = 0.08) and surgical resection (1.6 and 2.3 days for pre- and post-ACCESS groups respectively, p = 0.40) were similar.

**Conclusions:**

Patients admitted to ACCESS underwent more inpatient colonoscopies and were more likely to have definitive surgery on that admission. ACS services can facilitate the workup and management of complex surgical diseases such as emergency CRC without delaying treatment.

## Introduction

Colorectal cancer (CRC) is the third most commonly diagnosed cancer in the world, and one of the leading causes of cancer-related mortality [1]. Approximately fifteen to thirty percent of CRCs present as a surgical emergency, with the most common causes being obstruction, perforation, or bleeding [2,3]. Patients with emergency CRC may also present with metabolic, cardiovascular, infectious, or respiratory emergencies that significantly increase mortality [4]. Emergency surgery for CRC has been associated with poorer outcomes compared to elective surgery [2,3,5,6], and the increased morbidity and mortality of emergency CRC surgery places a significant clinical and financial burden on the hospital and the healthcare system [7-9].

Limitations in operating room (OR) resources may also hinder the expedited delivery of care for emergency patients [10,11]. Traditionally, on-call surgeons would either cancel their elective caseload to accommodate emergency surgeries, or delay operating on the emergency patient until they had elective OR time [12-14]. To mitigate this issue, acute care surgery (ACS) services have been widely adopted as a cost-effective model for delivering emergency surgical care [12-14]. ACS teams provide around-the-clock coverage to manage patients with all types of general surgical emergencies [14]. They have been shown to significantly reduce wait-times for urgent and emergent operations [15-18], expedite the efficient disposition of patients from the emergency room [15-18], and reduce hospital costs [11,16] without compromising patient care or safety [19].

However, the management of diseases which are commonly encountered by ACS services do not usually require long-term surveillance for disease recurrence [16,20]. The acute care of emergency CRC patients therefore presents a relatively more complex challenge as it requires the coordination of multiple specialties, including gastroenterologists, surgeons, and oncologists (medical and/or radiation) [2,3,5,8]. While ACS services in the United States are typically staffed by subspecialty trauma and acute care surgeons [19,20], many Canadian ACS teams are run by surgeons who also routinely perform cancer operations as part of their elective practices [14,21]. We, therefore, sought to assess whether the implementation of the Acute Care and Emergency Surgery Service (ACCESS) at our institution would expedite the surgical treatment of emergency CRC patients. Rather than assess the surgical management of emergency CRC *per se*, we elected to focus our study on the delivery of care for these patients.

## Methods

Ethics approval for this study was obtained through the Western University Research and Ethics Board (REB Number 102988). This study was conducted at the London Health Sciences Centre (LHSC), a tertiary-care hospital system with two university-affiliated institutions serving a metropolitan population of approximately 450,000. Additionally, the two centres receive referrals from 33 regional hospitals from 7 counties, covering a catchment area of 3 million [22]. Both hospitals within LHSC perform a high volume of colorectal cancer surgeries: University Hospital (UH), which lacks an ACS service (non-ACCESS), and Victoria Hospital (VH), where ACCESS was implemented in July 2010. The two sites function relatively independently, with no crossover of surgical consultants or gastroenterologists. At VH, all surgeons who participate in ACCESS also perform colorectal cancer operations as part of their elective practices.

We retrospectively reviewed adult (over 18 years old) patients who were admitted to LHSC after presenting to the Emergency Room (ER), and diagnosed with CRC. We compared patients whose care took place at VH between July 1, 2007 and June 30, 2010 (pre-ACCESS), and from July 1, 2010 to June 30, 2012 (post-ACCESS) as well as those treated at UH (non- ACCESS) from July 1, 2007 to June 30, 2012. The patients’ primary presenting complaints, reasons for admission, time to inpatient colonoscopy, and time to operative treatment were recorded. We assessed wait-times for inpatient endoscopy services (which are performed by gastroenterologists in both hospitals at LHSC) as a surrogate for examining the coordination of multiple specialties in the care of emergency CRC. We also reviewed characteristics of the malignancy such as the stage and tumour location, as well as patient outcomes, including disease-free and overall survival. Patients who underwent urgent diagnostic colonoscopy because of symptoms that suggested the presence of colon cancer (rectal bleeding, symptoms of obstruction, anemia, and weight loss) were considered to have had an inpatient colonoscopy if they were admitted for treatment within 48 hours of their colonoscopy. If patients were admitted to hospital more than 48 hours after their colonoscopy, they were considered to have had an outpatient colonoscopy. Because many of these patients had their colonoscopy at peripheral hospitals, or private endoscopy clinics outside of LHSC, we were unable to accurately ascertain the timing of their outpatient colonoscopy.

We excluded appendiceal neoplasms, carcinoid tumours, and goblet cell cancers since their management differs from the treatment of adenocarcinoma. We also excluded patients who had a previous history of CRC or inflammatory bowel disease as they undergo surveillance colonoscopy more frequently than the general population [23]. We also excluded patients who underwent colonic stenting, because of a lack of data pertaining to the placement of stents during the study period, and because of a lack of consensus regarding the use of stents in emergency CRC patients who are otherwise amenable to surgery [24,25].

Statistical analysis was performed using Graphpad Prism (Graphpad, La Jolla, California). Survival curves were compared by the Kaplan-Meier method. Continuous variables were compared between groups by Kruskal-Wallis one-way ANOVA with post hoc comparison between pre- and post-ACCESS groups by Dunn’s test [26]. Discontinuous variables were compared using Pearson chi-squared test. P values less than 0.05 were considered statistically significant.

## Results

We identified a total of 149 patients in our study: 47 (32%) were treated in the pre-ACCESS era; 37 (25%) patients were treated in the post-ACCESS era; and 65 (44%) patients were treated in the non-ACCESS hospital. There were no differences in the distribution of symptoms that led patients to present to the Emergency Department (p = 0.98): pain (70% of patients), altered bowel habits (54% of patients), and symptoms of obstruction (50% of patients) were the most frequently reported reasons for presenting to the emergency department (Table 1). Twenty two percent of patients (34 patients) presented with overt bleeding.

**Table 1:** Comparison of clinical characteristics among non-ACCESS, pre-ACCESS, and post-ACCESS groups at LHSC

**Clinical characteristics**	**Non-ACCESS**	**Pre-ACCESS**	**Post-ACCESS**	**P value**
Number of patients, n	65	47	37	-
Reason for presentation to hospital, n(%):				0.98
Change in bowel movements	40 (62)	26 (55)	14 (38)	
Rectal Bleeding	15 (23)	12 (26)	7 (19)	
Anemia	14 (22)	7 (15)	8 (22)	
Obstruction	37 (57)	22 (47)	15 (41)	
Pain	49 (75)	33 (70)	23 (62)	
Colonoscopy, n(%):				0.02
Prior outpatient colonoscopy	15 (23)	19 (40)	5 (14)	
Inpatient colonoscopy	16 (25)	9 (19)	14 (38)	
Indications for colonoscopy, n(%):				0.91
Change in bowel movements	15 (23)	5 (11)	15 (40)	
Rectal bleeding	13 (20)	7 (15)	11 (30)	
Anemia	14 (22)	6 (13)	7 (19)	
Obstruction	9 (14)	4 (8)	11 (30)	
Pain	17 (26)	9 (19)	15 (40)	
Location of malignancy, n(%):				0.49
Rectal	6 (9)	7 (15)	1 (3)	
Sigmoid and rectosigmoid	15 (23)	17 (36)	11 (30)	
Descending	6 (9)	4 (8)	3 (8)	
Transverse	7 (11)	4 (8)	3 (8)	
Ascending	31 (48)	15 (32)	19 (51)	
Stage, n(%):				0.15
0/I	3 (5)	5 (11)	4 (11)	
II	25 (38)	10 (21)	18 (49)	
III	25 (38)	20 (42)	11 (30)	
IV	10 (15)	9 (19)	4 (11)	
Unknown	2 (3)	4 (8)	0 (0)	

P values are shown for comparisons between pre- and post-ACCESS groups.

Seventy eight patients (52%) underwent colonoscopy: 31 patients (48%) were in the non-ACCESS group; 28 patients (60%) were in the pre-ACCESS group; and 19 patients (51%) were in the post-ACCESS group (Table 1). There were no statistical differences between the three groups for symptoms necessitating colonoscopy (p = 0.91), location of the malignancy (p = 0.49), or pathological stage (Table 1; p = 0.15). However, we observed a significant difference in the distribution of inpatient and outpatient colonoscopies between the pre- and post-ACCESS groups. In the pre-ACCESS group, 9 patients (19%) had an inpatient colonoscopy while 19 patients (40%) had an outpatient colonoscopy; in contrast, 14 post-ACCESS patients (38%) had an inpatient colonoscopy compared to only 5 patients (14%) who had an outpatient colonoscopy (p = 0.02).

We also observed a significant difference between the pre- and post-ACCESS groups with respect to the timing of surgical treatment following inpatient colonoscopy (Table 2). In the pre-ACCESS group, five out of 9 patients undergoing inpatient colonoscopy (56%) were discharged and underwent surgery during a separate admission: three patients were diagnosed with CRC after an admission for rectal bleeding, stabilized with blood transfusions, and underwent elective surgery within a week of being discharged from their initial admission, due to a lack of emergency OR time. Two additional patients were discharged following inpatient colonoscopy, and underwent coronary angiograms for optimization of their cardiovascular status and reduction of their perioperative morbidity. In contrast, 100% of patients in the post-ACCESS group had their surgery during the same admission as their colonoscopy (p = 0.006). In the non-ACCESS group, three patients (19%) were discharged following inpatient colonoscopy for rectal bleeding and were operated in separate admissions within one to two weeks after their initial admission.

**Table 2:** Comparison of outcomes between non-ACCESS, pre-ACCESS, and post-ACCESS groups at LHSC

**Characteristics**	**Non-ACCESS (*n*** **= 65)**	**Pre-ACCESS (*n*** **= 47)**	**Post-ACCESS (*n*** **= 37)**	**P Value**
Inpatient colonoscopy and surgery performed on same or separate admission, n(%):				0.006
Same admission	13 (20)	4 (8)	14 (38)	
Separate admission	3 (5)	5 (11)	0 (0)	
Median time from admission to inpatient colonoscopy, d (IQR^1^)	3.5 (2.4-6.9)	2 (0.9-3.6)	1.8 (1.3-3.1)	0.08
Median time from colonoscopy to OR, d (IQR^1^):	3.1 (0.3-8.5)	2.8 (1.0-4.0)	2.1 (1.2-2.5)	0.34
Same admission for colonoscopy and surgery	3.0 (0.14-3.6)	1.8 (0.3-4.0)	2.1 (1.2-2.5)	0.86
Separate admissions for colonoscopy and surgery	11.1 (9.0-12)	3.6 (2.8-11)	0 (0)	0.004
Median time from admission to OR, d (IQR^1^):	2.5 (0.93-45)	1.6 (0.8-4.6)	2.3 (1.1-4.6)	0.40
Without colonoscopy	1.4 (0.8-4.2)	1.6 (0.8-4.4)	1.5 (0.7-2.8)	0.89
With colonoscopy	6.6 (4.7-11.5)	4.4 (2.7-4.8)	4.5 (3.5-5.3)	0.87
Type of operation performed, n(%):				0.96
Primary anastomosis	49 (75)	35 (74)	27 (73)	
Ostomy	16 (25)	12 (26)	10 (27)	
Median length of stay, d (IQR^1^)	13.5 (8.8-19.2)	10.0 (6–17.2)	12 (8.5-18.5)	0.16
Status as of September 2012:				0.31
Disease-free	28 (43)	19 (40)	26 (70)	
Alive with disease	11 (17)	2 (5)	6 (16)	
Died of disease	18 (28)	19 (40)	3 (8)	
Died of other causes	8 (12)	7 (15)	2 (6)	

P values are shown for comparisons between pre- and post-ACCESS groups.

^1^IQR: Inter-quartile range (25%-75%).

Median wait-times from admission to inpatient colonoscopy were similar among the three groups (Table 2). Additionally, there were no differences in median wait-times from inpatient colonoscopy to surgery, if both were performed during the same admission (p = 0.86). When the inpatient colonoscopy and surgery were performed on separate admissions, however, we observed a significant difference in wait-times between the pre- and post-ACCESS groups (3.6 and 0 days respectively, p = 0.004). We did not observe any differences in hospital stay (p = 0.16), overall survival, or disease-free survival between the three groups of patients (Table 2).

## Discussion

The emergency presentation of CRC may be considered an extreme expression of the waiting time paradox where the outcomes are poor but the “waiting time” is very short [27]. Although many factors may contribute to the increased morbidity and mortality of emergency CRC patients compared to their elective counterparts [4,28-30], the presentation of these patients at night or on weekends to centres with reduced clinical services after-hours has been implicated as a potential contributor to mortality [28]. In this study, we demonstrate that an ACS service which provides around-the-clock emergency general surgery coverage expedites the in-hospital workup and treatment of emergency CRC patients within a single admission.

To date, many studies of ACS services have focussed on the delivery of care for patients presenting with acute appendicitis and cholecystitis, the two most frequently encountered diseases in acute care surgery [14-16,31]. Following an operation for these conditions, patients typically have a short hospital stay and limited outpatient follow-up. Emergency CRC therefore represents a more complex disease in the context of an ACS service, because its management requires the coordination of multiple aspects of care (diagnosis, workup, and treatment) provided by different medical and surgical specialties. Since most inpatient colonoscopies are performed by gastroenterologists at LHSC, we assessed inpatient endoscopy wait-times as a surrogate for the multidisciplinary coordination of care among emergency CRC patients. While a significant proportion of pre-ACCESS patients had received a colonoscopy as an outpatient, the implementation of ACCESS enabled a majority of emergency CRC patients to undergo inpatient colonoscopy after admission to hospital, and facilitated the performance of their surgery during the same admission. In contrast, more than half of all pre-ACCESS patients were discharged after their colonoscopy due to the lack of emergency operative time, and readmitted at a later date for elective surgery, with significantly increased wait-times as a consequence. Therefore, ACS services such as ACCESS may represent a model of high-value care [9,32], wherein the availability of dedicated ACS hospital beds and nursing staff, as well as the concentration of multiple procedures and operations within a single admission, facilitates the workup and treatment of emergency surgical patients in a timely and cost-effective manner [11,12,19,31].

Similar to other studies, 50% of patients presented with obstruction, while 22% presented with overt bleeding [6,33]. Interestingly, we did not observe the preponderance towards higher stages that previous studies have shown among patients with emergency CRC [29,30,34]. Among our population, only 15% of patients had distant metastases, compared to 25% in a retrospective study and 37% in a large prospective analysis [30,34]. Although select patients with metastatic CRC may benefit from a concurrent resection of the primary malignancy and liver metastases [35], coordination with a hepatobiliary surgeon may be challenging in emergency CRC due to time constraints. Moreover, the limited sample size in our study precludes an evaluation of outcomes, which may be resolved by a multi-centre analysis of emergency CRC patients operated by ACS services.

In our study of emergency CRC patients, 52% were diagnosed by colonoscopy, which is slightly higher than other studies [36]. Concerns about the median wait-times for inpatient endoscopy in emergency CRC patients mirror the concerns for outpatients with CRC [37]. While we observed a trend toward reduced wait-times for inpatient colonoscopy in the post-ACCESS group, future cost-benefit analyses are necessary to determine the ideal balance for allocating endoscopy resources towards outpatient and inpatient procedures within the constraints of a publicly-funded healthcare system [38].

In the absence of an ACS service with dedicated OR time, access to emergency OR resources may be affected by a multitude of factors, including competing surgical specialty access, consultant practice patterns, and the availability of anesthesiologists and other OR support staff [10,11,13,14,39]. The overall hospital length of stay was similar among the three groups, and comparable to other studies [33]. While we did not observe differences in patient outcomes, long-term follow-up of a large number of patients will be necessary to identify differences between groups. However, given that the biology of CRC tumours among emergency patients may be more aggressive and invasive compared to non-emergency or elective CRC patients [29], the expedited treatment of patients within a single admission, as demonstrated by our study, may play a role in improving clinical outcomes for emergency CRC.

As with the implementation of any new surgical service, the organization of ACCESS underwent subtle changes throughout the study period in order to optimize the utilization of operative resources. While we observed a longer, but statistically insignificant, wait-time between colonoscopy and surgery for post-ACCESS patients, a large prospective multi-centre analysis of institutions with ACS services may help identify more emergency CRC patients, determine their outcomes in and out of hospital, and highlight any potential inefficiency in the setup of ACS services with respect to wait-times for colonoscopies and surgeries.

There are several limitations in this study. Although endoscopy can be used to provide symptomatic relief for patients (including decompressing an acutely obstructed colon [40,41] or halting gastrointestinal bleeding), it was beyond the scope of our study to examine whether the colonoscopies were performed with therapeutic intent. Additionally, none of the patients in our study underwent colonic stenting. While its use as a bridge to elective surgery remains controversial in patients presenting with emergency CRC [24,25], future prospective cohort studies of all emergency CRC patients (surgical and non-surgical) are needed to assess the value of colonic stenting in this population. Additionally, we did not consider whether the surgeries were performed with curative or palliative intent, because it may not have been clearly evident at the time of the operation. Since pathology information—a necessity prior to referral to our regional cancer centre— was usually not available during the patients’ hospital stay, patients would be typically sent home and provided a referral to the cancer centre by the operating surgeon’s office once the pathology results were provided. Therefore, information regarding referral to adjunct services was not available for our study population.

Our study focuses on access to colonoscopic diagnosis of emergency CRC as a surrogate for multidisciplinary care. However, referral to other subspecialty services may potentially confound our analysis, especially if procedures are needed to optimize patients prior to surgery, such as placement of inferior vena cava filters (as prophylaxis to prevent pulmonary emboli), or performance of angiograms to diagnose and treat cardiovascular disease. We were also unable to obtain information regarding the number and timing of outpatient colonoscopies in our study population, because the procedures were often performed in community hospitals or private endoscopy clinics outside of our institution. This data would provide a true reflection of overall wait-times for surgical resection among emergency CRC patients, and could be addressed by a prospective analysis. While it is possible that patients who underwent colonoscopy may have presented to a peripheral facility for management of their emergency CRC (thereby underestimating estimates of the study population overall), we believe this is unlikely in most cases because these patients are typically transferred to LHSC, which serves as the regional cancer centre, for surgical management.

In conclusion, we demonstrate that the implementation of ACCESS expedites the treatment of emergency colorectal cancer patients by combining the diagnosis, workup, and surgical treatment within a single admission without delaying treatment. This study adds to the growing body of evidence that ACS programs effectively deliver surgical care, and can also potentially improve the quality of delivered care for patients who require more complex care. Although the availability of colonoscopy resources for emergency CRC patients is only one of many equally valid outcomes for CRC, our experience demonstrates that the reorganization of resources can significantly improve access to emergency colonoscopies for a vulnerable population. Future multi-centre studies examining the impact of ACS services on emergency cancer care are needed to demonstrate differences in clinical outcomes among this population.

## Competing interest

The authors do not declare any actual or potential conflicts of interest.

## Authors’ contributions

RVA designed the study, collected the data, performed the data analysis and drafted the manuscript. NP and KL helped to design the study. MB, NP, and KL provided critical revisions of the manuscript for important intellectual content. All authors approved the final version of the manuscript.
